# Apoptotic or Antiproliferative Activity of Natural Products against Keratinocytes for the Treatment of Psoriasis

**DOI:** 10.3390/ijms20102558

**Published:** 2019-05-24

**Authors:** Tse-Hung Huang, Chwan-Fwu Lin, Ahmed Alalaiwe, Shih-Chun Yang, Jia-You Fang

**Affiliations:** 1 Department of Traditional Chinese Medicine, Chang Gung Memorial Hospital, Keelung 204, Taiwan; huangtsehung@gmail.com; 2 School of Traditional Chinese Medicine, Chang Gung University, Kweishan, Taoyuan 333, Taiwan; 3 Graduate Institute of Health Industry Technology, Chang Gung University of Science and Technology, Kweishan, Taoyuan 333, Taiwan; 4 School of Nursing, National Taipei University of Nursing and Health Sciences, Taipei 112, Taiwan; 5 Department of Cosmetic Science, Chang Gung University of Science and Technology, Kweishan, Taoyuan 333, Taiwan; cflin@mail.cgust.edu.tw; 6 Research Center for Food and Cosmetic Safety and Research Center for Chinese Herbal Medicine, Chang Gung University of Science and Technology, Kweishan, Taoyuan 333, Taiwan; 7 Department of Anesthesiology, Chang Gung Memorial Hospital, Kweishan, Taoyuan 333, Taiwan; 8 Department of Pharmaceutics, College of Pharmacy, Prince Sattam Bin Abdulaziz University, Al Kharj 11942, Saudi Arabia; alalaiwe@gmail.com; 9 Department of Cosmetic Science, Providence University, Taichung 433, Taiwan; yangsc@pu.edu.tw; 10 Pharmaceutics Laboratory, Graduate Institute of Natural Products, Chang Gung University, Kweishan, Taoyuan 333, Taiwan; 11 Chinese Herbal Medicine Research Team, Healthy Aging Research Center, Chang Gung University, Kweishan, Taoyuan 333, Taiwan

**Keywords:** natural product, psoriasis, keratinocyte, apoptosis, proliferation, mechanism of action

## Abstract

Natural products or herbs can be used as an effective therapy for treating psoriasis, an autoimmune skin disease that involves keratinocyte overproliferation. It has been demonstrated that phytomedicine, which is used for psoriasis patients, provides some advantages, including natural sources, a lower risk of adverse effects, and the avoidance of dissatisfaction with conventional therapy. The herbal products’ structural diversity and multiple mechanisms of action have enabled the synergistic activity to mitigate psoriasis. In recent years, the concept of using natural products as antiproliferative agents in psoriasis treatment has attracted increasing attention in basic and clinical investigations. This review highlights the development of an apoptotic or antiproliferatic strategy for natural-product management in the treatment of psoriasis. We systematically introduce the concepts and molecular mechanisms of keratinocyte-proliferation inhibition by crude extracts or natural compounds that were isolated from natural resources, especially plants. Most of these studies focus on evaluation through an in vitro keratinocyte model and an in vivo psoriasis-like animal model. Topical delivery is the major route for the in vivo or clinical administration of these natural products. The potential use of antiproliferative phytomedicine on hyperproliferative keratinocytes suggests a way forward for generating advances in the field of psoriasis therapy.

## 1. Introduction

**Figure 1:**
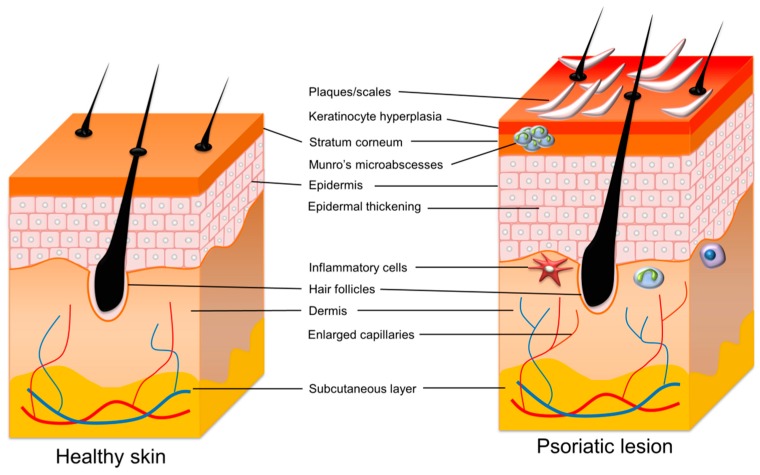
The comparison of healthy skin and psoriatic skin.

Psoriasis vulgaris is a genetic autoimmune disorder that manifests in the skin. Clinically, red plaques with silver or white multilayered scales characterize psoriasis, with a thickened acanthotic epidermis in patients who are markedly demarcated by adjacent nonlesional skin. Patients commonly report the symptoms of itching, pain sensation, and bleeding [1]. This disease has the features of high prevalence, chronicity, disfiguration, disability, and comorbidity [2]. Psoriasis can occur at any skin site, though it mostly features on the elbows and knees, back, trunk, and scalp. The fingernail and toenail regions are also often affected. The histopathological observation of psoriastic lesions reveals epidermal acanthosis, rete ridges, immune-cell infiltration in the dermis, and increased angiogenesis (Figure 1). The acanthosis is determined by keratinocyte proliferation that is associated with the altered differentiation procedure, as the maturation of keratinocytes occurs from the basal to the cornified layer [3]. Patients suffering from psoriasis are at risk of developing comorbid diseases. These include psoriatic arthritis, diabetes, cardiovascular disorders, Crohn’s disease, lymphoma, anxiety, and depression [4]. In addition, psoriasis always reduces the quality of life, and patients are exposed to social stigma and discrimination [5]. Psoriasis affects 2% to 3% of the population worldwide [6]. The prevalence of psoriasis has a median percentage of 1.43%, according to the global regional data [7]. Michalek et al. [8], summarizing the data from 20 countries, estimate that the prevalence of psoriasis in adults ranges from 0.5% to 11.4%, and in children from 0.1% to 1.4%. Psoriasis is equally prevalent in males and females, though a recent study demonstrates a more severe form in men than in women [9].

Psoriasis is an inflammatory skin disease that is derived from genetics, epigenetics, environments, and lifestyles. Different triggers, such as trauma, infection, drugs, and stress, can activate the immuno-inflammatory response in the skin, leading to excessive keratinocyte proliferation [10]. The initiation of psoriatic plaque starts with immune stimulation in susceptible subjects due to the exogenous triggers or the loss of immune tolerance via the recognition of autoantigens [11]. The antigens are presented to CD4^+^ and CD8^+^ T cells by dendritic cell (DC) subsets. Tumor necrosis factor (TNF)-α and interleukin (IL)-23 released by the activated DCs drive the polarization and clonal expansion of CD4^+^ and CD8^+^ IL-17- and IL-22-producing T lymphocytes, which results in the creation of a considerable amount of IL-17 and IL-22 in psoriatic lesions [12]. There are five types of psoriasis: plaque psoriasis, eruptive psoriasis, inverse psoriasis, pustular psoriasis, and erythrodermic psoriasis. The most common form is plaque psoriasis, with more than 80% of patients presenting erythematous scaly plaque [10]. Clinical finding, but not biopsy often, makes the diagnosis of psoriasis. Psoriasis Area and Severity Index (PASI), based on the presence of erythema, thickness, infiltration, scaling, and the extent of the lesions, quantify the severity of the disease [13]. The easy-to-use scores, such as the Psoriasis Global Assessment (PGA) and Lattice System-Physicians Global Assessment (LS-PGA), are also employed in routine clinical practice [14]. Various psoriasis-screening methods, including the Psoriatic Arthritis Screening and Evaluation (PASE) questionnaire, the Toronto Psoriatic Arthritis Screening (ToPAS) questionnaire, and the Psoriasis Epidemiology Screening Tool (PEST), are helpful with the diagnosis. The sensitivity of the three tools is reportedly similar (74.5%~76.6%) [15].

Although psoriasis has long been considered to be an immune-cell-dependent disease, keratinocytes’ critical role in inducing the early pathogenic events and sustaining the prolonged phase of the disorder cannot be ignored [16]. The hyperproliferation and abnormal differentiation as a secondary phenomenon elicited by the immune response is the pathogenic function of keratinocytes in psoriasis. Keratinocytes respond to the psoriatic lesions with an overactive wound-healing procedure. This process can produce epidermal thickening and expansion into the papillary dermis. Normally, the skin cells mature and shed from the cutaneous surface every 30 days. However, this maturation period shortens to three to six days, and it moves to the cutaneous surface as psoriasis develops [17]. The activated keratinocytes in psoriasis also play an important role in stimulating immune-cell infiltration. IL-1α that is derived from keratinocytes represents an additional inducer of innate immunity, favoring the migration of monocytes and neutrophils in the early stage of papule formation. The chemokine chemerin that is released by the keratinocytes is responsible for the accumulation of BDCA-2^+^ plasmacytoid DCs into early psoriatic skin [18]. Hence, the inhibition of keratinocyte proliferation or the promotion of keratinocyte apoptosis is a feasible approach to mitigating psoriatic lesions. Many patients have not responded to conventional psoriasis therapy, or, until now, even to the best available therapy. The use of complementary and alternative medicine (CAM) can be an efficient way to provide patients with multiple choices for psoriasis treatment. CAM includes dietary supplements, herbal therapy, traditional Chinese medicine, and mind intervention [19]. A preference for natural sources, the lower risk of adverse effects, and dissatisfaction with conventional therapy can be the reasons for which patients select CAM to treat psoriasis. In the epidermis, more than 90% of cells are keratinocytes. They are stratified into five layers: the stratum basale, spinosum, granulosum, lucidum, and corneum. Keratinocytes are an essential target for psoriasis treatments while using natural products. This review aimed to summarize the therapeutic efficacy and delivery/targeting potency of natural products, including crude extracts and pure compounds, on psoriasis vulgaris.

## 2. Molecular Pathogenesis of Psoriasis

Some exogenous triggers can lead to the occurrence of psoriasis. These nonspecific triggers include trauma, scratching, sunburn, and chemical irritants. Some drugs, such as lithium, non-steroidal anti-inflammatory drugs, β blockers, and antimalarials, are reported to exacerbate this disorder [20]. Occupational risk that impairs the nature of the cutaneous barrier subsequently aggravates psoriasis. Human immunodeficiency virus (HIV) is also a trigger for psoriasis. The HIV-infected patients with pre-existing psoriasis usually show a flare-up of lesions, which is complicated to manage [21]. Skin is a very complex organ with different cell types and neuroendocrine property for maintaining homeostasis [22]. Several cells in the skin are involved in the pathogenesis of psoriasis, with keratinocytes and immune cells being the major cell types related to psoriasis. Both of these can form a vicious psoriasis-producing cycle. Some autoantigens that are derived from keratinocytes, such as the LL37 cathelecidin/nucleic acid complex and the newly generated lipid antigen, are identified as launching the initial T cell activation, especially the subset of T lymphocyte-expressing IL-17A (Th17 cells). Once stimulated, the Th17 cells release the mediators, such as IL-17A, IL-17F, and IL-22, to elicit keratinocyte proliferation and inflammatory-marker production. Subsequently, the activated keratinocytes generate antimicrobial peptides, cytokines, and chemokines as chemoattractants for infiltrating the immune cells; this infiltration, in turn, amplifies the immune responses [23]. The chemoattractants that were derived from keratinocytes can activate the recruitment of plasmacytoid DCs, T cells, macrophages, and neutrophils to cause skin inflammation. The chronic inflammation within the skin also promotes skin aging [24,25].

DCs are the most potent antigen-presenting cells (APCs) of the immune system. There are significant increases of myeloid CD11c^+^ DCs in inflamed psoriatic skin. The plasmacytoid DCs are also largely present in psoriatic skin. The activated dermal DCs are important for initiating psoriatic plaque via the production of TNF, IL-12, and IL-23, and the excitation of the autoimmune CD4^+^ and CD8^+^ T cells [26]. Upon activation, the CD4^+^ and CD8^+^ T cells proliferate and migrate to the epidermis, where they recognize autoantigens and generate IL-17 and IL-22 [27]. Psoriatic lesions can be characterized by a marked accumulation of CD4^+^ T cells in the dermis and CD8^+^ T cells in the epidermis. Most of the epidermal CD8^+^ T cells express CD103, which is an integrin binding to E-cadherin to facilitate CD8^+^ T cell migration into the epidermis [28]. The naïve T cells also infiltrate the psoriatic lesions. Keratinocytes and APCs in the epidermis and dermis activate these cells, respectively. The cytokines contribute to expanding T cell infiltration by both modulating T cell proliferation and apoptosis and increasing the resistance of effector T cells to regulatory immunosuppression [29]. The plaque also comprises a large number of macrophages that secrete IL-6, IL-12, IL-23, and inducible nitric oxide synthase (iNOS). The macrophages have been proven to be the predominant source of TNF-α needed to initiate the inflammatory response for psoriasis-phenotype development [30]. Neutrophils are a characteristic component of lesional psoriatic skin. The activated neutrophils are recruited into the stratum corneum in the early stage and they create focal aggregates, called Munro’s microabscesses [31]. The enhancement of TNF-α and IL-17 signaling in the psoriatic plaque drives the neutrophil accumulation into the inflamed skin area, activating keratinocytes to release chemokine (C-X-C motif) ligand (CXCL)1, CXCL2, CXCL3, CXCL5, and IL-8 [32]. In turn, neutrophils produce reactive oxygen species (ROS), IL-17, cathelicidin, and neutrophil extracellular traps (NETs) as proinflammatory signals to reduce the inflammation.

**Figure 2:**
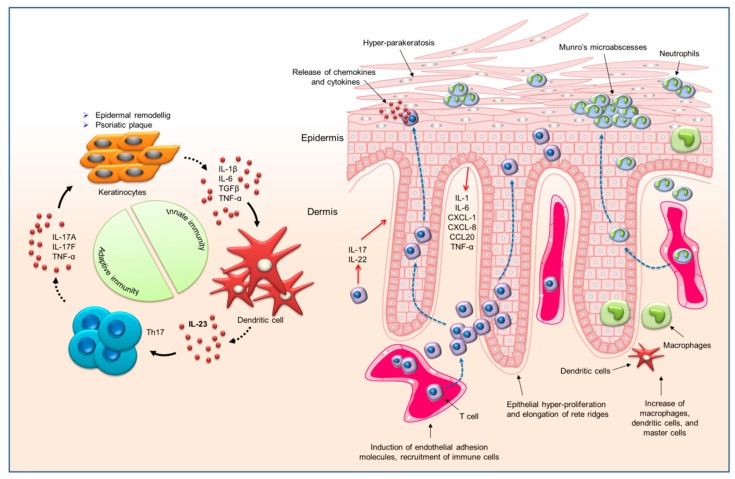
The cell types involved in psoriasis pathogenesis and the related pathways and interactions.

The TNF-α- and IL-23/Th17-dependent pathways are regarded as vital for psoriasis development. TNF-α is a pro-inflammatory cytokine that amplifies inflammation via several distinct pathways. This cytokine is produced through a number of cells, such as keratinocytes, lymphocytes, macrophages, and endothelial cells [33]. TNF-α increases the upregulation of adhesion molecules and secondary mediators, both of which are related to the development of psoriasis. Thus, the successful outcome of TNF-α’s blocking biologics in treating psoriasis is not surprising [34]. The exploration of the role of IL-17 and IL-23 in psoriasis pathogenesis has led to the understanding of immune events in this disease and a paradigm shift in the drug therapy. IL-23 largely acts on memory T lymphocytes, because the IL-23 receptor is absent in the naïve T cells. Other cytokines, such as IL-9, also support Th17-related inflammation. Under the regulation of IL-23, T lymphocytes that contain a high level of IL-17 form a self-amplifying and feed-forward inflammatory response in keratinocytes, which establishes a thickened epidermis with a cluster of immune-cell infiltration [35]. Figure 2 illustrates the mechanisms of psoriasis development at the cellular and molecular levels.

## 3. Therapeutic Drug Approaches for Psoriasis

Currently, the conventional therapies for psoriasis vulgaris can be divided into topical administration, systemic therapy, and biologics. The topical administration includes topical drugs and phototherapy. Some evidence-based guidelines are established for the management of psoriasis, including the North American guidelines, the International European guidelines, and the German S3 guidelines [2,36]. Approximately 70% of psoriasis patients present mild to moderate severity [37]. Topical treatment is recommended as the first-line therapy for the mild to moderate forms. The use of topical drugs in antipsoriatic therapy includes calcineurin inhibitors, steroids, vitamin D_3_ derivatives, retinoids, keratolytic agents, dithranol, and coal tar. The selection of topical treatment depends on the patient’s needs, the type of psoriasis, the lesional site, the cosmetic acceptability, and the duration that is available for application [38]. Topical calcineurin inhibitors, such as tacrolimus and pimecrolimus, are employed for the sites that are difficult to treat, e.g., the intertriginous region and the face. Potent corticosteroids are ideal for scalp treatment, which is also difficult. Vitamin D_3_ analogs, such as calcipotriol, calcitriol, and tacalcitol, are effective in treating psoriasis via the normalization of keratinocyte proliferation and differentiation. In addition, the vitamin D_3_ analogs inhibit the inflammatory response by modulating the immune events [39]. A combination of topical corticosteroids and vitamin D_3_ analogs has reportedly shown a synergistically effective treatment of psoriasis [40]. The keratolytic agents include salicylic acid, lactic acid, and urea. They disrupt intra-keratinocyte adhesion in the uppermost layer of the epidermis to promote physiological shedding [41]. Phototherapy, such as ultraviolet B irradiation and psoralens plus ultraviolet A exposure (PUVA), can be classified as topical therapy for psoriasis. Photochemotherapy is one of the oldest therapies for psoriasis employing PUVA. It is useful for treating psoriasis due to the direct antiproliferative effect on keratinocytes [42]. The psoralens bind to the DNA, in which pyrimidine bases are the targets for photochemical apoptosis [43]. Table 1 summarizes the conventional therapeutic approaches for psoriasis treatment via topical application.

The systemic drugs are another choice for achieving efficient psoriasis treatment. Patients with the moderate or severe form or associated psoriatic arthritis, and those who do not adequately respond to topical therapy, may be treated with systemic drugs, such as cyclosporin, methotrexate, retinoids, and fumarates [44]. Caution should be taken, because severe side effects can complicate systemic therapy. For example, methotrexate demonstrates the risk of hepatotoxicity. With appropriate monitoring, systemic therapy can be used for the maintenance treatment of psoriasis. Selective phosphodiesterase (PDE)4 inhibitors have gained great attention for their anti-inflammatory activity in neutrophils in the treatment of psoriasis [45]. PDE4 inhibitors are reported to reduce neutrophil infiltration and hyperkeratosis in psoriasis [46]. Apremilast is a new systemic agent of PDE4 inhibitor that is approved for oral administration to treat psoriasis.

Increasing knowledge regarding the molecular pathogenesis of psoriasis has hastened the development of targeted therapy for psoriasis with monoclonal antibodies. These biologics target cytokines or specific inflammatory pathways, such as TNF-α, IL-17, and IL-23, to achieve highly selective immune suppression [47]. The biologics reveal superior therapy efficiency when compared to the other classes of antipsoriatic agents for patients with severe psoriasis. The biologic therapy has proven to be highly effective in improving psoriasis in 80% to 90% of patients [12]. TNF-α inhibitors etanercept, adalimumab, and infliximab are approved for moderate-to-severe psoriasis management. Secukinumab, ixekizumab, and brodalumab are the antibodies targeting IL-17 for the treatment of psoriasis. The antibody guselkumab is directed against IL-23. Ustekinumab is an antibody targeting p40 subunit that IL-12 and IL-23 share. All of these antibodies are used for long-term psoriasis treatment with negligible evidence of cumulative toxicity and drug interaction [48]. The combination of biologics with other drug therapy allows for dosage reduction, side-effect minimization, maintenance of initial response of biologics, and acceleration of the response to biologics [49]. For example, the efficacy of methotrexate against psoriasis is increased with greater safety when used in combination with biologics [50]. A major concern that is associated with using biologics is the possible risk of developing nonmelanoma skin cancer [51]. Monoclonal antibodies should be avoided in patients with active malignancy.

## 4. The Trend in Using Natural Products as Antipsoriatic Agents

Although most of the conventional therapies can reduce the symptoms of psoriasis, this disease has no known cure. Moreover, many therapies cause side effects, such as atrophy, organ toxicity, immunosuppression, infection, and carcinogenesis, which lead to the limitation of long-term use. It is necessary to develop alternative treatments for psoriasis to achieve the aims of superior effectiveness and fewer side effects. Natural medicine has gained much attention in the search for novel therapies. Natural products possess a richness of resources containing potentially bioactive compounds [52]. Herbal medicine is preferable for patients because it is safe. The herbal products’ structural diversity and multiple mechanisms of action have led to the synergistic activity that mitigates psoriasis. The herbal drugs can also increase the bioavailability due to the possibility of the presence of permeation enhancers inside the phytomedicine. The two topicals that are most frequently used by psoriasis patients in North and South America are reportedly steroids (16% to 79%) and CAM (10% to 62%) [53]. The utilization of CAM in the psoriasis patient population ranges from 39% to 62% in Asia and the Middle East [54]. Damevska et al. [55] reported that 47% of patients in South Europe use CAM as an antipsoriatic remedy. All of these data encourage the investigators to find the antipsoriatic agents from the traditional phytomedicine. Some clinical trials have been conducted by employing natural products to examine the effect of psoriasis mitigation. The meta-analysis of the clinical trials demonstrates that aloe vera, indigo naturalis, kukui nut oil, *Mahonia aquifolium*, and capsaicin are the most efficacious topical phytomedicines for treating psoriasis [56,57]. These natural products can ameliorate psoriatic lesions via the molecular mechanisms that are related to apoptosis, angiogenesis inhibition, and inflammation suppression [58].

Aloe vera is a perennial succulent plant that belongs to the *Liliaceae* family. Anthraquinones, polysaccharides, vitamins, and salicylic acid are the active ingredients of aloe vera exhibiting anti-inflammatory and anti-pruitic activities [59]. Topical indigo naturalis ointment is effective in reducing the PASI of psoriasis patients due to the anti-inflammatory and antiproliferative activities of indirubin in this extract [60]. Kukui nut oil, which is rich in polyunsaturated fatty acids, especially oleic acid, linoleic acid, and linolenic acid, displays an anti-inflammatory effect [61]. *Mahonia aquifolium*, well known as Oregon grape, belongs to the *Berberidaceae* family. The extract of *Mahonia aquifolium* contains the primary active agent of berberine, which is an isoquinoline alkaloid that inhibits hyperproliferation and inflammation in psoriatic lesions [62]. Substance P is sensitive in the case of psoriatic lesions in stimulating inflammatory cells to induce keratinocyte proliferation, vasodilation, and angiogenesis. Capsaicin can activate substance P due tothe affinity to vanilloid receptors, and it then depletes the cutaneous sensory neurons of substance P. This feature improves the redness and pruritus in psoriasis patients [63].

## 5. The Apoptotic or Antiproliferative Strategy to Ameliorate Psoriasis

**Figure 3:**
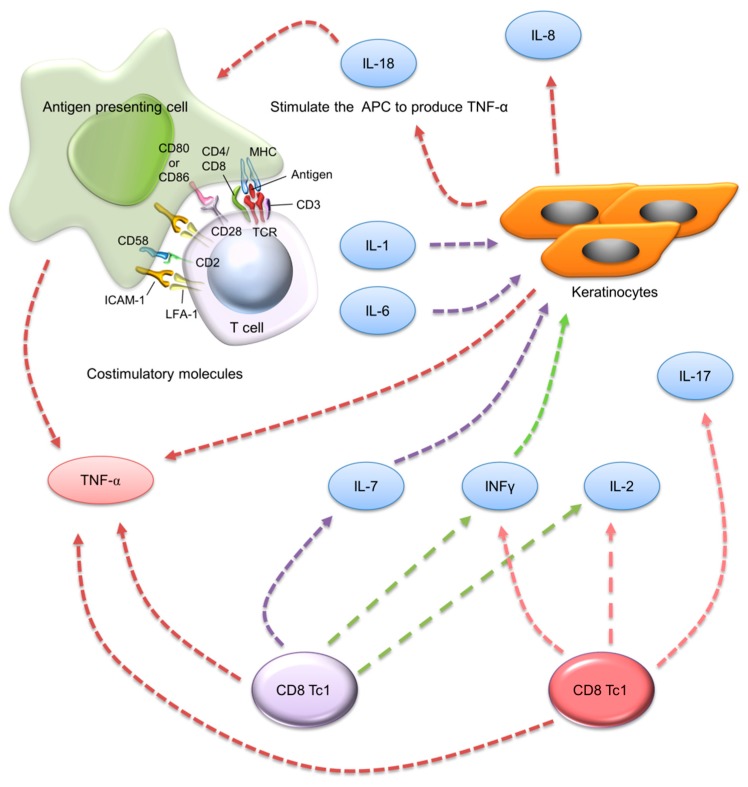
The apoptotic mechanisms of keratinocytes in psoriatic lesion.

It is supposed that the pathogenic pathways mainly involve keratinocytes in the beginning of psoriasis development. Upon activation by some triggers, such as mild trauma and pathogens, keratinocytes become a source of innate immune mediators [64]. In the chronic stage, the activation of DCs and effector T cells in the lesions establishes definite cytokines, which TNF-α, IL-17, IL-22, and interferon (IFN)-γ mainly represent. Keratinocytes contain cytokine receptors and potently respond by further releasing cytokines. The keratinocytes exhibit altered proliferation and differentiation under the impact of these cytokines [17]. The homeostasis between proliferation and differentiation is disrupted in psoriasis. The increased epidermal proliferation markers, such as Ki-67 and the proliferating cell nuclear antigen (PCNA), and the reduced differentiation markers, such as keratin 10, can describe the psoriatic plaque [65]. An increased resistance to apoptosis is also observed in the activated keratinocytes [66]. The keratinocyte proliferation that is induced by the cytokines contributes to thickened skin, a scaly surface appearance, epidermal hyperplasia, hyperkeratosis, and parakeratosis. The imbalance between proliferation and differentiation becomes a self-amplifying cycle, where the cytokines and altered homeostasis act on the immune cells to perpetuate the inflammatory response. An idea agent for treating psoriasis should have the role in antiproliferation, anti-inflammation, and immunomodulation. Melatonin is an example, which is a natural hormone with the integration of proliferation and inflammation suppression in the activated keratinocytes [67,68,69]. Figure 3 shows the apoptotic mechanisms of keratinocytes in the psoriatic lesion.

The keratinocyte-proliferation inhibition, modulation of keratinocyte differentiation, and apoptosis are been considered to be the therapeutic targets of psoriasis inhibition for both approved drugs and unapproved phytomedicines [70]. The prescribed antipsoriatic drugs, such as dithranol, vitamin D_3_ derivatives, and methotrexate, exhibit the therapeutic effect through restraining keratinocyte hyperproliferation or regulating keratinocyte differentiation. Among these agents, the vitamin D_3_ analogs are the most commonly used clinically. The topically applied vitamin D_3_ analogs can arrest the hyperproliferation of keratinocytes. Vitamin D_3_ acts chiefly on the vitamin D receptor to regulate cell growth, differentiation, and immune function, as well as calcium and phosphorus metabolism [71]. The established phototherapies for psoriasis include narrowband UVB and PUVA. Phototherapy is one of the most efficient options for treating psoriasis. Apoptosis and immune suppression are the predominant mechanisms of action for diminishing psoriatic lesions by phototherapy [72]. PUVA induces apoptosis through the creation of ROS to impair the cellular, mitochondrial, and nuclear membranes. PUVA also directly causes linkage of psoralen to pyrimidine bases, leading to DNA-synthesis inhibition [73]. Besides inducing apoptosis in keratinocytes, phototherapy also induces apoptosis in T cells and Langerhans cells [74]. Although phototherapy is very effective for psoriasis treatment, its time-consuming nature and short-term control of the disease have limited its application. The possibility of carcinogenesis of PUVA also restricts its long-term use. The unpleasant side effects and practical difficulty for psoriasis patients limit the topical use of dithranol and vitamin D_3_ derivatives. The search for new antiproliferative agents with low toxicity and an effective outcome remains urgent. Natural products provide an abundant resource for achieving this goal.

## 6. Natural Products for Antiproliferation against Keratinocytes

The recent application of natural medicine derived from plants confirms the capability to exert an apoptotic or antiproliferative effect on keratinocytes. The use of natural products ameliorates the symptoms of psoriasis vulgaris. Some natural products have been approved for clinical use or they are under clinical trial for preventive or therapeutic use against psoriasis. In addition, some herbal formulations and natural compounds are approved for managing psoriasis in cell-based and animal studies. The following describes the different therapeutic approaches to using natural products against psoriasis through apoptosis and/or hyperproliferation inhibition. We divided the natural products into two classes: crude extracts and pure compounds.

### 6.1. Crude Extracts for Treating Hyperproliferation of Psoriasis

The crude extracts from the natural resources under discussion are the concentrates derived from plant or animal sources. There can be different compounds in the crude extracts showing bioactivity. The combination of these bioactive agents in the extracts may be beneficial for displaying the synergistic effect for treating diseases. The usefulness of crude extracts in treating hyperproliferation of psoriasis has been investigated. Most of these extracts have been used to treat inflammatory diseases in traditional or folk medicine. Traditional Chinese medicine is extensively employed with high effectiveness and safety in the treatment of psoriasis. Tse et al. [75] investigated the antiproliferative effect of 60 Chinese medicinal materials, which are prescribed in Chinese medicine practice for psoriasis management, on keratinocytes (HaCaT), in vitro. These medicinal materials were extracted with 80% ethanol/water for a 3-(4,5-cimethylthiazol-2-yl)-2,5-diphenyl tetrazolium bromide (MTT) assay. Three materials, *Rubia cordifolia*, realgar, and *Coptis chinensis*, were effective in showing antiproliferative potency with an IC_50_ of 1.4, 6.6, and 23.4 μg/mL, respectively. Nevertheless, the realgar extract induced a modest inhibition of dermal fibroblast (Hs-68) growth with an IC_50_ of 48.1 μg/mL, which demonstrated cytotoxicity against normal cells. Tse et al. [76] further explored the molecular mechanisms of antiproliferative activity against HaCaT by *R*. *cordifolia*. The IC_50_ were >62.5, 18.3, 11.9, 5.8, 1.4, and 2.9 μg/mL for the treatment of *R*. *cordifolia* extract by 3, 6, 12, 24, 48, and 72 h, respectively. The percentage of keratinocytes in the sub-G1 phase increased from 0.3% to 15.2% following the increased concentration from 1 to 32 μg/mL. DNA fragmentation was found by gel electrophoresis and TUNEL. *R*. *cordifolia* could also activate caspase-3 expression. This evidence suggests that the induction of apoptosis was responsible for the antiproliferation that was stimulated by *R*. *cordifolia*.

Some polyphenols that are rich in the bark of forest trees are effective in the treatment of psoriasis [77]. García-Pérez et al. [78] examined the in vitro antiprorative activity of Canadian wood species in the growth of primary normal and psoriatic human keratinocytes. The bark of yellow birch (*Betula alleganiensis*), black spruce (*Picea mariana*), balsam fir (*Abies balsamea*), and jack pine (*Pinus banksiana*) trees was extracted while using 90% ethanol and hot water to obtain polyphenol-rich materials. The extracts of yellow birch and black spruce bark showed higher proliferation suppression than the others. The yellow birch extract at 90 μg/mL inhibited normal keratinocytes by 26%, but failed to affect psoriatic keratinocyte growth. The black spruce extract at 110 μg/mL inhibited the normal and psoriatic keratinocytes by 18% and 21%, respectively. The antiproliferative activity could be due to the presence of proanthocyanidins and hydroxycinnamic acids in the bark extracts. Huaier (*Trametes robiniophila*) is a fungus with potential as an antitumor agent through cell apoptosis [79]. Su et al. [80] evaluated the role of huaier in the treatment of psoriasis via the methodology of in vitro HaCaT growth inhibition. Huaier extract reduced HaCaT viability in a time- and concentration-dependent fashion. The extract concentration at 4, 8, and 16 mg/mL produced a cell viability percentage of about 60%, 40%, and 30%, respectively. The extract also blocked the cell cycle in the G1 phase, which indicated an apoptosis pathway. The oral huaier administration for four weeks efficiently reduced patients’ PASI score by 50%. Shraibom et al. [81] prepared a polyberbal formulation containing *Rheum palmatum*, *Lonicera japonica*, and *Rehmannia glutinosa* (1:1:3) to evaluate in vitro antipsoriatic activity. Chlorogenic acid, acteoside, and rhein were the major compounds in this formulation. The polyherbal formulation inhibited keratinocyte proliferation and elicited apoptosis. The formulation induced an increase in the early and late apoptotic cell percentage by 22- and 52-fold as compared to the control, respectively. DNA fragmentation increased by 1.8-fold when compared to the control cells. The downregulation of pro-inflammatory markers, such as IFN-γ, TNF-α, and IL-6, was also observed.

*Malva sylvestris* leaf is a medicinal herb that exerts the bioactivity of suppressing inflammation, gastric ulcers, and skin disorders [82]. It is proven to show anti-inflammatory activity in the acute skin inflammation animal model due to the main active compound of malvidin 3-glucoside [83]. Prudente et al. [84] further assessed the effect of the hydroalcoholic extract of *M*. *sylvestris* on hyperproliferation via in vitro HaCaT and in vivo 12-O-Tetradecanoylphorbol-13-acetate (TPA)-induced inflammation models. The cell viability was reduced by 36% and 91% at extract concentrations of 10 and 100 μg/mL, respectively. The cell-cycle profile confirmed the decreased proliferation by apoptosis. In vivo topical application of *M*. *sylvestris* caused an oedema reduction of 65%. The extract produced a decline of PCNA-positive cells by 66%, while the positive control (dexamethasone) showed a reduction of 92%. *Artemisia capillaris* is a phytomedicine that contains chlorogenic acids, coumarins, and flavonoids as the actives to exhibit therapeutic potential against cancers, hepatitis, malaria, obesity, and pathogen infection [85]. This herb has been proven to ameliorate atopic dermatitis-like lesions in Nc/Nga mice [86]. The therapeutic potential of the alcohol extract of *A*. *capillaris* was examined in HaCaT cells and in the imiquimod (IMQ)-induced psoriasis-like mouse model [87]. The IC_50_ of *A*. *capillaris* extract was 37.5 μg/mL after 72-h incubation. The caspase-3 activity in HaCaT was 1.9-fold higher in the treatment group than in the nontreatment control. The epidermal thickness could be reduced by 55% when compared to the vehicle control after the topical application of *A*. *capillaris* (50 mg/mL) in the IMQ-treated mouse for four days. The expression of the hyperproliferative marker Ki-67 was also decreased by *A*. *capillaris*. A problematic alcoholic extract application on the skin is a toxicity-related concern. Lee et al. [88] developed a cream formulation of *A*. *capillaris* for convenient and safe use. The cream base containing *A*. *capillaris* lowered the erythema and scaling of the psoriasis-like lesion in the in vivo IMQ-induced model. The histology demonstrated a 49% reduction in the epidermal thickness by using the cream compared to the control group.

Some clinical trials were conducted to evaluate the possibility of the application of herbal materials for psoriasis therapy. The in vitro data verified the ability of the bark extract that was derived from *Mahonia aquifolium* to inhibit HaCaT growth with an IC_50_ of 35 μM based on the alkaloid content with respect to berberine [89]. A psoriatic patient clinical trial was performed to compare the antipsoriatic effect of *M*. *aquifolium* ointment and dithranol [90]. A half-body randomized controlled trial was used to assess the hyperproliferation markers (keratin 6, keratin 15, and Ki-67) and the adhesion molecules (intercellular adhesion molecule-1) in biopsy. Both of the treatments diminished epidermal and dermal T cell infiltration, with less inhibition by *M*. *aquifolium*. Lin et al. [91] estimated the efficacy and safety of topically applied indigo naturalis (*Baphicacanthus cusia*) ointment on psoriasis. Indigo naturalis has been utilized for treating various inflammatory conditions and dermatosis in traditional Chinese medicine. Indigo naturalis and its active ingredient indirubin reveal antitumor activity that is involved in the suppression of cancer cell proliferation [92]. In the eight-week trial, in which 14 patients were enrolled, a significant reduction in the clinical score was obtained after the topical administration of indigo naturalis. The biopsy assay showed a marked decrease of Ki-67 and inflammatory marker CD3. The efficacy of the indigo naturalis ointment could be mediated by proliferation and differentiation modulation of the epidermal keratinocytes. *Curcuma xanthorrhiza* is a traditional herbal medicine that belongs to the *Zingiberaceae* family. HaCaT treated with *C*. *xanthorrhiza* extract demonstrates the capability of inhibiting IL-6, IL-8, and keratinocyte proliferation [93]. A 1% *C*. *xanthorrhiza* ointment was topically applied on 17 psoriatic patients using a double-blinded randomized trial [94]. *C*. *xanthorrhiza* significantly reduced the PASI score after topical application for four weeks. However, K6 expression showed no significant difference between the treatment and the placebo groups.

St. John’s wort (*Hypericum perforatum*) is traditionally used for the treatment of burns and diarrhea, and as a diuretic. This extract is topically applied to treat wounds, sunburns, ulcers, keloid scars, and hemorrhoids [95]. Two clinical trials verified the efficacy of St. John’s wort for psoriasis therapy. Ten patients with plaque-type psoriasis were enrolled for treatment with St. John’s wort [96]. The St. John’s wort ointment and the vehicle were topically applied on different sites of each patient twice daily. In estimating the PASI score, the St. John’s wort ointment, as compared to the placebo, significantly lowered redness, scaling, and thickness. The PASI for the extract and placebo was 1.8~2.1 and 0.7~1.1, respectively. It was found that St. John’s wort effectively mediated the TNF-α-induced apoptosis in keratinocytes in vitro [97]. Mansouri et al. [98] investigated the effect of topically applied St. John’s wort on TNF-α expression through a double-blind placebo-controlled trial in 20 psoriatic patients. The histological observation revealed a marked reduction of acanthosis, parakeratosis, Munro’s microabscesses, and spongiosis with the treatment using St. John’s wort ointment. The TNF-α level in the epidermis, basal layer, and dendritic cells was lessened from 0.58, 1.58, and 0.66 pg/mL to 0.16, 0.92, and 0.25 pg/mL, respectively. Table 2 summarizes the profiles for the antipsoriatic activity of different crude extracts with the aim of inhibiting keratinocyte proliferation.

### 6.2. Pure Compounds for Treating Hyperproliferation of Psoriasis

The components in crude extracts are usually complex. The difficulty of quality control is a disadvantage of using herbal products for medicinal application. Moreover, some ingredients in the extracts show no or negligible bioactivity. The crude extracts can be further fractionated and separated to exclude the useless parts and acquire the pure compounds with higher bioactivity. This concept approximates the development of drugs. There are many compounds that are derived from the crude extracts for preventing hyperproliferation in psoriasis. Resveratrol is a stilbene polyphenol from grapes and *Polygonum cuspidatum*. It is well known to present strong anti-inflammatory, anticancer, anti-diabetes, and antioxidant activities [99]. Holian and Walter [100] examined the antiproliferative activity of resveratrol against primary keratinocytes. Resveratrol produced a time- and concentration-dependent proliferation inhibition. The IC_50_ that was detected by hemocytomer cell count was 0.5 μM. The modulation on the cellular redox state by resveratrol contributed to the antiproliferative effect. Wu et al. [101] further elucidated the role of aquaporin 3 on the antiproliferative mechanism of resveratrol. Aquaporin 3 is a water-transporting protein that is expressed in epidermal keratinocytes. The overexpression of aquaporin 3 leads to keratinocyte proliferation and epidermal thickening [102]. The nontoxic concentrations (<40 μM) of resveratrol restrained the proliferation of the primary culture of neonatal human keratinocytes. Resveratrol at 40 μM significantly decreased the aquaporin 3 mRNA level >5-fold. This compound also inhibited the phosphorylation of extracellular signal-regulated kinase (ERK).

Curcumin that is derived from *Curcuma longa* is another active demonstrating an antiproliferative effect on keratinocytes. Curcumin is reported to show anti-inflammation, anticancer, and antioxidant properties [103]. Sun et al. [104] investigated the impact of curcumin on apoptosis of TNF-α-activated HaCaT cells. The expression of anti-apoptotic proteins, including the inhibitor of apoptosis (IAP)1, IAP2, and B-cell lymphoma-extra large (Bcl-xL), was enhanced by TNF-α, but inhibited by 7.37 μg/mL curcumin. Curcumin also inhibited TNF-α-activated NF-κB, IL-6, and IL-8. The antiproliferative ability of resveratrol can be enhanced in combination with light irradiation. The HaCaT cells were pretreated with curcumin at 0.1~1 μg/mL for 1 h and then irradiated with UVA or visible light [105]. The result revealed that the combination of curcumin (1 μg/mL) and UVA (1 J/cm^2^) induced 40% of the cells with apoptotic nuclei. This was a much higher percentage than in the group without UVA (0.5%). Cytochrome c released from the mitochondria, caspases-8 and -9 activation, and NF-κB inhibition represented the induction of apoptosis. Niu et al. [106] investigated the combination of curcumin, red light (630 nm), and blue light (405 nm) for attenuating the proliferation of TNF-α-activated HaCaT, a simulation of psoriasis lesions. The curcumin concentration at 0.16~2.5 μM showed no proliferation inhibition on karatinocytes. A significant inhibition was observed in the presence of 0.16 and 0.62 μM curcumin when it was combined with red light and blue light, respectively. The light alone showed no effect on proliferation. This indicated that the light exposure amplified the apoptosis of curcumin-treated keratinocytes. The combined treatment inhibited NF-κB activity and stimulated caspases-8 and -9 with the preservation of cell-membrane integrity.

Rottlerin is a polyphenol that is purified from *Mallotus phillippinensis*. This compound is reported to exert antihypertensive, antifertility, and antiallergic actions [107]. A previous study [108] demonstrated that rottlerin could block the cancer cell proliferation via the downregulation of cyclin D1 and the inhibition of NF-κB activity. Rottlerin is also a potent suppressor of keratinocyte proliferation through the prevention of basal and hydrogen-peroxide-stimulated NF-κB elevation [109]. Min et al. [110] investigated the inhibitory effect of rottlerin on primary keratinocyte proliferation. The apoptotic percentage of keratinocytes following treatment with rottlerin at 5 and 10 μM was 27% and 56%, respectively. The levels of TNF-α, IL-6, and IL-23 were significantly reduced in the TPA-activated keratinocytes after rottlerin treatment. The oral rottlerin also relieved the IMQ-stimulated psoriasiform lesion by suppressing keratinocyte proliferation, immune-cell infiltration, and vascular proliferation. Acridone alkaloids are natural compounds that are purified from the *Rutaceae* family. Many synthetic analogs are developed to evaluate the antitumor effect, because they can inhibit cell growth [111]. The 10-substituted hydroxy-10H-acridin-9-ones are the synthetic derivatives of acridone and they can be regarded as aza-analogs of the antipsoriatic drug dithranol. Putic et al. [112] prepared a series of 10-substituted hydroxy-10H-acridin-9-ones to assess the antiproliferative potency against HaCaT. The compounds with benzyl substitution at the 10-position showed greater growth inhibition than the other substitutions. The most potent compound was the analog possessing N-methyl moiety and 1,3-dihydroxy arrangement at the acridone scaffold. The IC_50_ of this compound (0.6 μM) was similar to that of dithranol (0.7 μM). The extract of *R*. *cordifolia* was proven to induce the apoptosis of psoriasis-relevant HaCaT cells [76]. 1,4-Dihydroxy-2-naphthoic acid (DHNA) is an anthraquinone precursor that is present in *R*. *cordifolia* extract. The possible application of DHNA in psoriasis was tested in an in vitro HaCaT model [113]. The IC_50_ of DHNA (38.9 μg/mL) was higher than that of dithranol (9.4 μg/mL) after 72-h treatment. On the other hand, the cytotoxicity against skin fibroblasts was minor for DHNA when compared to dithranol. This may lead to the conclusion that the use of DHNA is safe. This compound elicited keratinocyte apoptosis via G0/G1 cell-cycle arrest.

Arsenic-containing minerals are considered as therapeutic materials for topical use in the treatment of scabies, carbuncles, herpes zoster, and psoriasis [114]. Three inorganic arsenics were tested to check whether they presented antiproliferative activity on HaCaT [115]. Arsenic trioxide (As_2_O_3_), arsenic pentoxide (As_2_O_5_), and arsenic iodide (AsI_3_) displayed keratinocyte growth inhibition with IC_50_ of 2.4, 16.0, and 6.8 μM, respectively. These molecules showed a moderate inhibition of fibroblast viability with the IC_50_ of 43.4~223.0 μM. All of the arsenics produced DNA fragmentation and caspase-3 activation. Quercetin, a naturally occurring flavonoid, was reported to assist the keratinocyte-growth inhibition that is induced by arsenic trioxide [116]. Quercetin induced apoptosis through ROS generation in different cell systems, including MOLT-4 cells, leukemia cells, and MCF-7 cells [117]. The combined arsenic trioxide and quercetin led to keratinocyte apoptosis by ROS-related p53 protein ubiquitination. The apoptotic markers, such as caspase-3, DNA ladders, and poly (ADP-ribose) polymerase (PARP), were increased by the combination with a decrease of mitochondrial membrane potential. However, the application of arsenic for psoriasis therapy may be hindered due to the possibility of arsenic toxicity inducing skin carcinoma [118]. Baicalein is another flavonoid that regulates keratinocyte differentiation and proliferation [119]. The treatment of HaCaT with baicalein (10 μM) slightly inhibited the proliferation without affecting ROS production, cytochrome c, and apoptosis. Baicalein treatment increased the cell population in the G_0_/G_1_ phase from 60% to 70%. Baicalein also increased the expression of keratins 1 and 10, which are the indicators of cell differentiation.

Celastrol is a triterpene isolated from *Celastrus orbiculatus*. The extract of *C*. *orbiculatus* has long been used in Chinese medicine to treat bacterial infection, rheumatoid arthritis, and skin diseases. Zhou et al. [120] evaluated the antiproliferative effect of celastrol on HaCaT cells and primary human keratinocytes. Celastrol inhibited the growth of HaCaT and primary keratinocytes in a concentration-dependent manner, with an IC_50_ of 1.1 and 2.9 μM, respectively. The IC_50_ for fibroblasts was 6.8 μM. Celastrol significantly downregulated anti-apoptotic Bcl-2 expression and increased the pro-apoptotic Bcl-2-associated X protein (Bax), which suggests the involvement of both death-receptor and mitochondrial pathways in the apoptotic process. *Salvia miltiorrhiza* extract is employed in treating psoriasis, atopic dermatitis, and inflammation-related diseases [121]. Tanshinone IIA is the major constituent of *S*. *miltiorrhiza* extract. Li et al. [122] examined the possibility of using tanshinone IIA for keratinocyte apoptosis in connection with exploring the treatment mechanism of psoriasis. The IC_50_ on the growth of mouse keratinocytes incubated for 24, 48, and 72 h was 4.3, 1.9, and 1.1 μg/mL, respectively. The apoptosis led to S phase arrest, accompanied by a decrease in cyclin A and phospho-cyclin-dependent kinase 2 (pCdk2). A caspase pathway was also involved in the keratinocyte apoptosis. Dehydrocostuslactone and costunolide are sesquiterpene lactones that were isolated from certain plants, such as *Magnolia sieboldii*. Both of the compounds are effective in mitigating inflammation and exerting pro-apoptotic activity [123,124]. Scarponi et al. [125] explored the effect of both naturally occurring lactones on the regulation of inflammation and proliferation of keratinocytes to cytokines. The lactones suppressed proliferation and cell-cycle progression-related gene expression, as the compounds enhanced cell-cycle arrest and apoptosis. Dehydrocostuslactone and costunolide decreased the inflammatory and regulatory genes in IL-22-activated keratinocytes, including C-C motif chemokine Ligand 2 (CCL2), C-X-C motif chemokine 10 (CXCL10), and intercellular adhesion molecule 1 (ICAM-1). These findings encourage the further application of psoriasis therapy.

Delphinidin is an anthocyanidin that provides the primary plant pigment and it serves as an antioxidant. Delphinidin has been determined as a possible treatment for psoriasis, because it can inhibit inflammation and regulate keratinocyte differentiation [126]. A three-dimensional (3D) reconstructed human psoriasis skin equivalent was employed as a model to investigate the change in the proliferation and inflammation by delphinidin [127]. A prolonged treatment of delphinidin at 20 μM on the skin equivalent for five days greatly inhibited the expression of the proliferation biomarkers Ki-67 and PCNA. Delphinidin also inhibited inflammatory biomarkers, including iNOS, S100A7-psoriasin, and S100A15-koebnerisin, which are usually found in psoriatic lesions. *Rhodomyrtus tomentosa* is a plant that belongs to the *Myrtaceae* family. The leaves of *R*. *tomentosa* have been traditionally used to treat a number of diseases, including diarrhea, infection, and open wounds. Rhodomyrtone is a major active of *R*. *tomentosa* extract, which demonstrates antibacterial, anti-inflammatory, and immunomodulatory properties [128]. Chorachoo et al. [129] investigated whether rhodomyrtone could affect the proliferation, growth arrest, and apoptosis of HaCaT. The antiproliferative percentage of HaCaT was 13.6%~61.6% after 24-h incubation with rhodomyrtone at 2~32 μg/mL. In the scratching assay, rhodomyrtone at 2 μg/mL delayed the wound closure by 61.8%. Chromatin condensation and nuclei fragmentation were detected after rhodomyrtone treatment. The flow cytometry demonstrated an elevation of the apoptotic percentage when compared to the control. An in vivo skin irritation test in a rabbit showed no redness or oedema after rhodomyrtone administration. The inhibitory effect of rhodomyrtone on TNF-α/IL-17A-driven inflammation was further rated [130]. Rhodomyrtone decreased the inflammatory gene expression in the human skin organ culture. This molecule also inhibited TNF-α-activated ERK, JNK, and p38 phosphorylation. The topical delivery of rhodomyrtone (0.18 and 0.64 mg/cm^2^) on an IMQ-induced psoriasiform lesion in a mouse effectively decreased the epidermal thickness and hyperplasia. Taken together, both antiproliferation and anti-inflammation are the therapeutic mechanisms for psoriasis inhibition by rhodomyrtone.

Phytosphingosine is found in plants, fungus, and the human epidermis. Phytosphingosine and its derivatives are known to prevent water loss from the skin, as well as to regulate epidermal cell growth, apoptosis, and differentiation [131]. Kim et al. [132] synthesized two phytosphingosine derivatives, (Z)-4-oxo-4-(((2S,3S,4R)-1,3,4-trihydroxyoctadecan-2-yl)amino)but-2-enoic acid (mYG-II-6) and (E)-4-oxo-4-(((2S,3S,4R)-1,3,4-trihydroxyoctadecan-2-yl)amino)but-2-enoic acid (fYG-II-6), to investigate the anti-inflammatory and antipsoriatic activity. Both of the derivatives enhanced the expression of the pro-apoptotic markers Bax and Bcl-2-associated death promoter (Bad), as well as caspase 3. TPA induced in vivo skin inflammation in mice. Both of the topically applied compounds effectively suppressed the inflammatory responses via the inhibition of mitogen-activated protein kinase (MAPK), NF-κB, and Janus kinase/signal transducer of activation (JAK/STAT) signaling. Lower toxicity was observed for the derivatives than for phytosphingosine. Amentoflavone is a biflavonoid that possesses anti-inflammatory and antioxidant effects [133]. A possibility for the application of amentoflavone for psoriasis treatment was examined [134]. This biflavonoid inhibited proliferation and promoted apoptosis with the decreased cyclin D1, IL-17A, and IL-22 in cytokine-stimulated HaCaT. In the in vivo IMQ-induced psoriasis model, oral amentoflavone (50 mg/kg) decreased the epidermal thickness from about 300 to 180 μm. This compound significantly downregulated a series of cytokines (IL-17A, IL-22, and IL-23) in the lesion. Zhang et al. [135] screened 250 Chinese medicine compounds for keratinocyte growth suppression and found that periplogenin exhibited the greatest ability to induce apoptosis. The IC50 for periplogenin was 1.56 μg/mL. Periplogenin is a cardenolide from *Aegle marmelos* leaves. The authors further performed the antiproliferative mechanism and the in vivo psoriasis-like hyperplasia model. The results indicated that this cardenolide elicited necroptotic cell death through oxidative stress. The ear thickness and the weight of the IMQ-treated mouse were decreased by periplogenin. The histological visualization showed a reduced hyperplasia and inflammatory cell infiltration by topically applied periplogenin.

Recently, Horinouchi et al. [136] investigated the anti-proliferative and anti-inflammatory effects of 3β,6β,16β-trihydroxylup-20(29)-ene (TTHL) for the possible development on psoriasis treatment. This compound belongs to a triterpene structure that was purified from the *Combretum leprosum* flower, a folk medicine in Brazil for treating skin disorders. A previous study [137] suggests antiproliferative activity of TTHL against cancer cells through apoptosis and ROS generation. This mechanism was also shown in the case of keratinocytes. TTHL at 10 μM reduced keratinocyte viability to 90%. Some 95% of keratinocytes entered the sub-G1 phase, which denoted apoptotic cells. TTHL with an ED_50_ of 0.328 μmol/ear inhibited the increase of the ear thickness by TPA. The neutrophil marker, myeloperoxidase, of the TPA-treated ear was reduced to the normal baseline by 1.3 μmol/ear TTHL. This compound decreased PCNA-positive cells by 72%, which was comparable to the positive control dexamethasone. Rhododendrin is an arylbutanoid glycoside that was isolated from *Rhododendron brachycarpum*. It is an inhibitor of inflammation for use in treating inflammatory skin diseases [138,139]. Jeon et al. [140] elucidated the detailed action mechanism of this compound and its relevance as a therapy for psoriasis. The in vitro keratinocyte study demonstrated the inhibition of the toll-like receptor (TLR)-7/NF-κB and MAPK pathways by rhododendrin. Caveolin-1 loss in keratinocytes contributes to psoriasis development. The caveolin-1 expression was preserved with TLR-7 upon treatment with rhododendrin, which indicated a critical role of this compound in maintaining skin homeostasis. Topical rhododendrin delivery significantly reduced IMQ-induced hyperplasia, immune-cell infiltration, and cytokines (TNF-α, IL-1, IL-6, IL-8, IL-17, and IL-23).

8-methoxypsoralen (8-MOP) is commonly used in PUVA therapy for treating psoriasis through keratinocyte-proliferation constraint. The chemical structure of 8-MOP, which is largely found in *Psoralea corylifolia*, is classified as furocoumarin. *P*. *corylifolia* extract for topical application is beneficial in mitigating psoriatic-lesion eczema and alopecia [141]. Some analogs of 8-MOP exist in *P*. *corylifolia*. Alalaiwe et al. [142] as compared the skin absorption and antipsoriatic activity of 8-MOP and its derivatives. 8-MOP, isopsoralen, and bakuchiol showed a comparable pig skin absorption of 0.47, 0.58, and 0.50 nmol/mg, which was greater than that of psoralen (0.25 nmol/mg) and psoralidin (0.14 nmol/mg). The combination of UVA with 8-MOP or isopsoralen led to greater proliferation inhibition than the other compounds. The concentration at 0.25 μM could completely restrain keratinocyte growth. The topical application of PUVA on IMQ-treated mouse skin demonstrated a decrease in the epidermal thickness from 117 to 62 and 26 μm by 8-MOP and isopsoralen, respectively. PUVA also depressed Ki-67-positive cells in the epidermis. However, PUVA, which is used in clinics, has a risk of causing squamous-cell cancer [143]. Epigallocatechin-3-gallate (EGCG) is a tea polyphenol that could reduce the risk of cancer associated with PUVA and promote the normal differentiation of keratinocytes [144]. Zhang et al. [144] investigated whether EGCG could inhibit IMQ-stimulated psoriasiform lesions. The topical delivery of EGCG for six consecutive days decreased the epidermal thickness from 70~150 to 30~80 μm. This could be due to the capability of EGCG to reduce PCNA expression and enhance the caspase 14 level. The involvement of terminal differentiation and normal barrier formation is the predominant role of caspase 14 on keratinocytes. Table 3 summarizes the profiles for antipsoriatic activity of the natural compounds htat aimed to suppress keratinocyte proliferation.

## 7. Conclusions

The current therapeutic options for psoriasis patients have some disadvantages, which include frustration with medication efficacy, inconvenience, time constraints, and possible adverse effects. Until now, an effective and long-term regimen for psoriasis eradication has been lacking, especially for moderate to severe psoriasis. There is a great need for the continuous development of novel, safe, and effective treatment modalities for psoriasis. Among many active compounds that were investigated for psoriasis mitigation, phytochemicals derived from natural resources have become of great interest over the last decades. Several investigations assessing natural-sources-based psoriasis therapy reveal potential activity, especially the antiproliferative effect. Until now, most of the reports concerning the antiproliferative efficacy of natural compounds for psoriasis treatment have been based on laboratory or animal-related work. Some of the investigations suggest the usefulness of natural products for psoriasis treatment, only for their ability to suppress keratinocyte proliferation. The evidence from in vitro cell study and in vivo animal tests offers limited information regarding the natural products’ clinical success. Convincing results in human clinical studies are urgently needed to encourage future investigation on this topic. Scientists should pay attention, not only to the therapeutic benefits of natural products, but also to their adverse effects on human health. Caution should be used in optimizing the feasible conditions of phytomedicine to balance the effectiveness of psoriasis therapy and tissue damage or toxicity.

## Figures and Tables

**Table 1:** Current topical drug therapy for psoriasis treatment.

Drug	Pharmacological Mechanisms	Side Effects on Skin
Calcineurin inhibitors	Inhibition of T cell activation and of pro-inflammatory cytokine synthesis	Itching and stinging
Glucocorticosteroids	Anti-inflammation, anti-mitosis, apoptosis, vasoconstriction, and immunomodulation	Skin atrophy after long term use
Vitamin D_3_ derivatives	Regulation of keratinocyte proliferation, differentiation and apoptosis	Stinging, burning, and peeling skin
Retinoids	Normalization of keratinocyte proliferation and differentiation	Redness, peeling, dryness, itching, and burning sensation
Keratolytics	Softening/hydration of the stratum corneum and desquamation of hyperkeratotic skin	Redness, swelling, tenderness, and pustules
Dithranol	Inhibition of keratinocyte hyperproliferation, granulocyte function, and immune response	Redness and irritation
Coal tar	Inhibition of keratinocyte proliferation and correction of the defect of keratinocyte differentiation	Redness, burning, itching, and skin staining
Ultraviolet B irradiation (UVB)	Alteration of cytokine profile, induction of apoptosis, and promotion of immunosuppression	Burning and itching
Psoralens plus ultraviolet A exposure (PUVA)	Inhibition of DNA replication and production of cell cycle arrest, alteration in the expression of cytokines	A risk for skin cancer

**Table 2:** Crude extracts derived from the plants for treating hyperproliferation of psoriasis.

Plant	Experimental Model	Cell or Animal	Method for Detecting Proliferation	Outcomes Offered by Extract	Reference
60 Chinese herbal medicines	In vitro	HaCaT	MTT assay	*Rubia cordifolia*, realgar, and *Coptis chinensis* showed high antiproliferative effect	Tse et al. [75]
*Rubia cordifolia*	In vitro	HaCaT	MTT and TUNEL	Apoptosis is the main mechanism for antiproliferation of HaCaT	Tse et al. [76]
Canadian wood species	In vitro	Normal and psoriatic human keratinocytes	MTT and trypan blue	Yellow birch and black spruce showed high antiproliferative effect	García-Pérez et al. [78]
Huaier	In vitro	HaCaT	CASY cell counting	A significant proliferation inhibition through apoptosis pathway	Su et al. [80]
*Rheum palmatum*, *Lonicera japonica*, and *Rehmannia glutinosa*	In vitro	HaCaT	Annexin-V staining and caspase-3	Antiproliferative and anti-inflammatory activities	Shraibom et al. [81]
*Malva sylvestris*	In vitro/in vivo	HaCaT/Swiss mouse	MTT assay/TPA-induced inflammation	Inhibition of HaCaT proliferation and oedema caused by TPA	Prudente et al. [83]
*Artemisia capillaris*	In vitro/in vivo	HaCaT/Balb/c mouse	Annexin-V staining/IMQ-induced lesion	Inhibition of HaCaT proliferation and epidermal thickness caused by IMQ	Lee et al. [87]
*Artemisia capillaris*	In vivo	Balb/c mouse	IMQ-induced lesion	Reduction of epidermal thickness caused by IMQ	Lee et al. [88]
*Mahonia aquifolium*	Clinical	Psoriatic patients	Biopsy	Reduction of epidermal and dermal T cell infiltration	Augustin et al. [90]
Indigo naturalis (*Baphicacanthus cusia*)	Clinical	Psoriatic patients	Clinical score and biopsy	Reduced Ki-67 and CD3	Lin et al. [91]
*Curcuma xanthorrhiza*	Clnical	Psoriatic patients	Clinical score and K6 expression	Reduced PASI score	Rahmayunita et al. [94]
St. John’s wort (*Hypericum perforatum*)	Clnical	Psoriatic patients	Clinical score	Reduced PASI score	Najafizadeh et al. [96]
St. John’s wort (*Hypericum perforatum*)	Clnical	Psoriatic patients	TNF-α expression	Reduced TNF-α in epidermis and dendritic cells	Mansouri et al. [98]

IMQ, imiquimod; MTT, 3-(4,5-cimethylthiazol-2-yl)-2,5-diphenyl tetrazolium bromide; TPA, 12-O-Tetradecanoylphorbol-13-acetate; TUNEL, terminal deoxynucleotidyl transferase dUTP nick end labeling.

**Table 3:** Pure compounds derived from the plants for treating hyperproliferation of psoriasis.

Compound	Experimental Model	Cell or Animal	Method for Detecting Proliferation	Outcomes Offered by Extract	Reference
Resveratrol	In vitro	Primary culture of human keratinocytes	MTT assay and hemocytometer cell counting	Dose-dependent antiproliferative activity	Holian and Walter [100]
Resveratrol	In vitro	Primary culture of human keratinocytes	7-aminoactinomycin D assay	Reduced aquaporin 3 expression	Wu et al. [101]
Curcumin	In vitro	TNF-α-activated HaCaT	Annexin-V staining	Apoptosis is the main mechanism for antiproliferation of HaCaT	Sun et al. [104]
Curcumin	In vitro	HaCaT	Nuclei staining	Increased apoptosis by the combination with UVA or visible light	Dujic et al. [105]
Curcumin	In vitro	TNF-α-activated HaCaT	Cell counting kit-8 and LDH assay	Increased apoptosis by the combination with red and blue light	Niu et al. [106]
Rottlerin	In vitro	Primary culture of human keratinocytes	MTT assay	Increased apoptosis in an autophagy-dependent pathway	Min et al. [110]
Acridone analogs	In vitro	HaCaT	Cell counting and LDH assay	The compounds with benzyl substitution at the 10-position showed potent proliferation inhibition	Putic et al. [112]
1,4-Dihydroxy-2-naphthoic acid	In vitro	HaCaT	Sulphorhodamine B staining	Dose-dependent antiproliferative activity	Mok et al. [113]
Arsenics	In vitro	HaCaT	MTT assay	Significant inhibition on keratinocyte growth with moderate toxicity on fibroblasts	Tse et al. [115]
Arsenic trioxide	In vitro	HaCaT	MTT and LDH assay	Increased apoptosis via ROS-dependent p53 ubiquitination	Shen et al. [116]
Baicalein	In vitro	HaCaT	Cell counting	Minor inhibition of proliferation without affecting ROS production	Huang et al. [119]
Celastrol	In vitro	HaCaT and primary human keratinocytes	MTT assay	Increased apoptosis via Bcl-2 attenuation and Bax upregulation	Zhou et al. [120]
Tanshinone IIA	In vitro	Primary mouse keratinocytes	MTT assay	Increased apoptosis via caspase pathway	Li et al. [122]
Dehydrocostuslactone and costunolide	In vitro	Primary human keratinocytes	Annexin-V staining	Inhibited proliferation and inflammation-related genes	Scarponi et al. [125]
Delphinidin	In vitro	3D reconstructed psoriatic skin equivalent	Ki-67 and PCNA	Inhibited proliferation and inflammation	Chamcheu et al. [126]
Rhodomyrtone	In vitro/in vivo	HaCaT/rabbit	MTT assay	Elevated apoptosis with no skin irritation	Chorachoo et al. [129]
Rhodomyrtone	In vitro/in vivo	Human skin organ culture/ICR mouse	Histology	Reduced epidermal thickness and hyperplasia	Chorachoo et al. [130]
Phytosphingosine derivatives	In vitro/in vivo	HaCaT/hairless mouse	Cell viability assay kit	Increased programmed keratinocyte death	Kim et al. [132]
Amentoflavone	In vitro/in vivo	HaCaT/Balb/c mouse	Cell counting kit	Inhibited proliferation and epidermal thickness	An et al. [134]
Periplogenin	In vitro/in vivo	HaCaT/Balb/c mouse	MTT assay	Inhibited proliferation via necroptotic cell death	Zhang et al. [135]
3β,6β,16β-Trihydroxylup-20(29)-ene	In vitro/in vivo	HaCaT/Swiss mouse	MTT and neutral red assay	Reduced keratinocyte viability via apoptosis and ROS generation	Horinouchi et al. [136]
Rhododendrin	In vitro/in vivo	Normal human keratinocytes/C57BL/6 mouse	MTT assay	Inhibited proliferation and epidermal thickness	Jeon et al. [139]
8-Methoxypsoralen derivatives	In vitro/in vivo	HaCaT/Balb/c mouse	MTT assay	Inhibited proliferation and epidermal thickness	Alalaiwe et al. [142]
Epigallocatechin-3-gallate	In vivo	Balb/c mouse	PCNA	Inhibited epidermal thickness and differentiation regulation	Zhang et al. [144]

Bax, Bcl-2-associated X protein; LDH, lactate dehydrogenase; MTT, 3-(4,5-cimethylthiazol-2-yl)-2,5-diphenyl tetrazolium bromide; PCNA, proliferating cell nuclear antigen; ROS, reactive oxygen species.
